# Applications of Tissue Decellularization Techniques in Ventricular Myocardial Biofabrication

**DOI:** 10.3389/fbioe.2022.802283

**Published:** 2022-02-21

**Authors:** Aravind Krishnan, Hanjay Wang, John Ward MacArthur

**Affiliations:** Department of Cardiothoracic Surgery, Stanford University School of Medicine, Stanford, CA, United States

**Keywords:** cardiomyopathy, biofabrication, decellularization, stem cells, transplant surgery

## Abstract

Ischemic heart disease is the leading cause of death around the world, and though the advent of coronary revascularization has revolutionized its treatment, many patients who sustain ischemic injury to the heart will go on to develop heart failure. Biofabrication of ventricular myocardium for replacement of irreversibly damaged ischemic myocardium is sought after as a potential therapy for ischemic heart failure, though challenges in reliably producing this biomaterial have limited its clinical application. One method that shows promise for generation of functional myocardium is the use of tissue decellularization to serve as a scaffold for biofabrication. This review outlines the methods, materials, challenges, and prospects of tissue decellularization techniques for ventricular myocardium biofabrication. Decellularization aims to preserve the architecture and composition of the extracellular matrix of the tissue it is applied to, allowing for the subsequent implantation of stem cells of the desired cell type. Decellularization can be achieved with multiple reagents, most of which have detergent properties. A variety of cell types can be implanted in the resulting scaffold, including cardiac progenitor cells, and embryonic or induced pluripotent stem cells to generate a range of tissue, from patches to beating myocardium. The future of this biofabrication method will likely emphasize patient specific tissue engineering to generate complex 3-dimensional constructs that can replace dysfunctional cardiac structures.

## Introduction

Ischemic heart disease remains the number one cause of mortality around the world (Virani et al., 2021). Though coronary revascularization via percutaneous coronary intervention or coronary artery bypass grafting has revolutionized its treatment, myocardial infarction will often progress to heart failure, and a subset of patients will experience end-stage heart failure (Bourassa et al., 1993; Mosterd and Hoes, 2007; King et al., 2010; Briceno et al., 2016; Velazquez et al., 2016; Bahit et al., 2018; Sun et al., 2018; Sun et al., 2020).

While orthotopic heart transplantation is the gold standard treatment for end-stage heart failure, it is challenged by limited donor supply, the lifelong need for immunosuppression, and the need to monitor for rejection (Heidenreich et al., 2020; Bozkurt et al., 2021). Though the use of mechanical circulatory support with left ventricular assist devices or extracorporeal membrane oxygenation, and more aggressive transplantation strategies have increased utilization and improved outcomes in patients awaiting heart transplantation, there is still a huge unmet clinical need for organ replacement (Mehra et al., 2019; Guenthart et al., 2021). Tissue engineered bioscaffolds have long been proposed as adjunctive therapies that may address heart failure by restoring or replacing the damaged architecture in failing hearts or even the whole heart (Arenas-Herrera et al., 2013; Daley et al., 2018; Tang-Quan et al., 2018).

The two prevailing approaches to generating bioscaffolds for myocardial repair or replacement are “bottom-up” and “top-down” engineering (Arenas-Herrera et al., 2013; Tang-Quan et al., 2018). The “bottom-up” approach focuses on bioprinting scaffolds from native or engineered extracellular matrix components with or without colocalized progenitor cell lines that differentiate into myocardium (Ong et al., 2018). The “top-down” approach produces bioscaffolds from decellularization of tissues or whole organs, leaving acellular extracellular matrix and retained tissue architecture, onto which stem cell lines are then implanted or reperfused, eventually producing functional tissues/organs (Arenas-Herrera et al., 2013; Tang-Quan et al., 2018). The proposed benefit of this latter approach is in the retention of vascular networks through which a recellularized tissue structure could then be perfused (Badylak, 2002; Pla-Palacín et al., 2018; Taylor et al., 2018).

Herein, we will briefly review the history and prevalent methods of decellularization as a method of biofabrication of myocardial tissue, and then focus on its application to replacing ventricular myocardium.

## Decellularization and Recellularization

### History

Tissue decellularization is the process by which chemical, enzymatic, or mechanical stimuli are used to produce an acellular bioscaffold from previously cellularized tissue or whole organs (Wainwright et al., 2010; Gilpin and Yang, 2017; Iop et al., 2017; Rana et al., 2017; Saldin et al., 2017; Daley et al., 2018; Spang and Christman, 2018; Tang-Quan et al., 2018). Prior to 2008, decellularization had mainly been applied to tissues without complex vascular networks; cardiac tissue decellularization had been limited to valve tissue and portions of the aorta (Badylak, 2004; Ketchedjian et al., 2005; Gilbert et al., 2006). In 2008, whole heart decellularization was introduced by the Taylor group whereby cadaveric rat hearts were decellularized through coronary perfusion of a detergent, retaining their coronary vascular networks, and then recellularized via a direct injection method supported by perfusion of cell media through the coronary vasculature (Ott et al., 2008). This method produced hearts that macroscopically contracted, producing stroke work up to 25% of a fetal heart’s function (Ott et al., 2008). The subsequent decade produced a convention in determining decellularization in that the decellularized tissue must meet a threshold of <50 ng of double stranded DNA per mg of dry weight and <200 base pairs of DNA fragment length (Nagata et al., 2010). Further improvements in decellularization materials and methods improved the degree of decellularization of whole hearts, and introduced decellularization and reperfusion of porcine hearts (Akhyari et al., 2011; Remlinger et al., 2012; Momtahan et al., 2015). In 2016, the Ott group introduced decellularization and recellularization of human hearts deemed unsuitable for transplantation (Guyette et al., 2016).

#### Contemporary Methods of Decellularization

Conventional approaches to decellularization of tissue include bathing the tissue in a decellularization agent (Methe et al., 2014) or, in the case of cardiac tissue, intracoronary perfusion of a chemical or enzymatic decellularization agent (Tang-Quan et al., 2018). Chemical decellularization is most commonly achieved with a detergent agent such as sodium dodecyl sulfate (SDS), which can be used in conjunction with other detergents such as Triton X-100 in optimal concentrations to preserve the architecture of the extracellular matrix (Ott et al., 2008; Cebotari et al., 2010; Keane et al., 2015; Gilpin and Yang, 2017).

Enzymatic decellularization involves the use of nucleases such as Dornase or trypsin meant to cleave RNA and DNA or attachments from cells to the ECM, to achieve the standard thresholds of decellularization (Lynch and Ahearne, 2013). This method is most frequently used in conjunction with chemical decellularization but risks inadvertent damage to the extracellular matrix itself (Crapo et al., 2011). Viability of the decellularized scaffold is directly dependent on preservation of the ECM and microvascular architecture (Taylor et al., 2018).

Decellularization can be achieved using physical agitation or alternating cycles of freezing and cooling, or pressurization with supercritical carbon dioxide that lyse the cells (Tang-Quan et al., 2018). Each of these methodologies can be used in combination with other methods, and ultimately a group’s protocol for decellularization results from trialing multiple methods to achieve an acceptably low level of remaining DNA.

#### Recellularization

Decellularized tissues can subsequently be seeded with stem cells via a process called recellularization. The decellularized tissue can be stereotropically repopulated with vascular tissue, muscle, and other functional components of organized myocardium (Tang-Quan et al., 2018). Recellularization with cardiac cell lines can be applied to decellularized ECM (dECM) or bio-scaffolds that are not cardiac in origin (Tan et al., 2009; Francisco et al., 2020). Methods of recellularization are largely based on perfusion of stem cells, direct injection of stem cells, or a combination of the two, into decellularized ECM (Ng et al., 2011; Guyette et al., 2016; Tang-Quan et al., 2018).

Induced pluripotent stem cells (iPSCs) and mesenchymal stem cells (MSCs) are the most commonly used recellularization agents, and differentiate into the appropriate functional unit of the area into which they are seeded due to biomechanical cues from the extracellular matrix (ECM) (Ott et al., 2008; Hynes, 2009; Bowers et al., 2010; Schmuck et al., 2018). Perfusion based recellularization in particular takes advantage of this cross-talk that occurs between the cell line and the ECM by utilizing pauses in perfusion where the perfused cells are able to form physical attachments to the ECM, while simultaneously delivering a critical mass of stem cells to engraft the decellularized tissue (Ott et al., 2008; Hülsmann et al., 2013; Tang-Quan et al., 2018). Direct injection, on the other hand, suffers from poor engraftment, commonly with fewer than 50% of injected cells appropriately seeding the tissue (Iop et al., 2017). The two approaches can be used in tandem to maximize both spatial specificity and diffuse engraftment of recellularized cell populations, as demonstrated by Weymann et al. who utilized perfusion human umbilical cord blood endothelial cells through the coronaries of decellularized porcine hearts, followed by direct injection of rat neonatal cardiomyocytes into the ventricular wall to ultimately generate electrically responsive, viable recellularized porcine hearts (Weymann et al., 2014).

Advances in recellularization have centered on improvements in engraftment as well as specific programming of iPSCs into complex cell lines that form the components of functional myocardium (Iop et al., 2017). A major milestone in successful recellularization is evidence of the formation of gap junctions, allowing for coordinated conductivity, and ultimately, beating myocardium (Tang-Quan et al., 2018). Despite these advances, whole organ recellularization is still limited in efficacy. Attempts at performing heterotopic heart transplantation with decellularized and subsequently recellularized hearts in pigs showed non-viable hearts with non-patent coronary arteries or poorly functional left ventricular myocardium (Kitahara et al., 2016). Despite these limitations, decellularized bioscaffolds presently hold great potential to augment or replace ventricular myocardium.

## Ventricular Augmentation and Biofabrication

**FIGURE 1:**
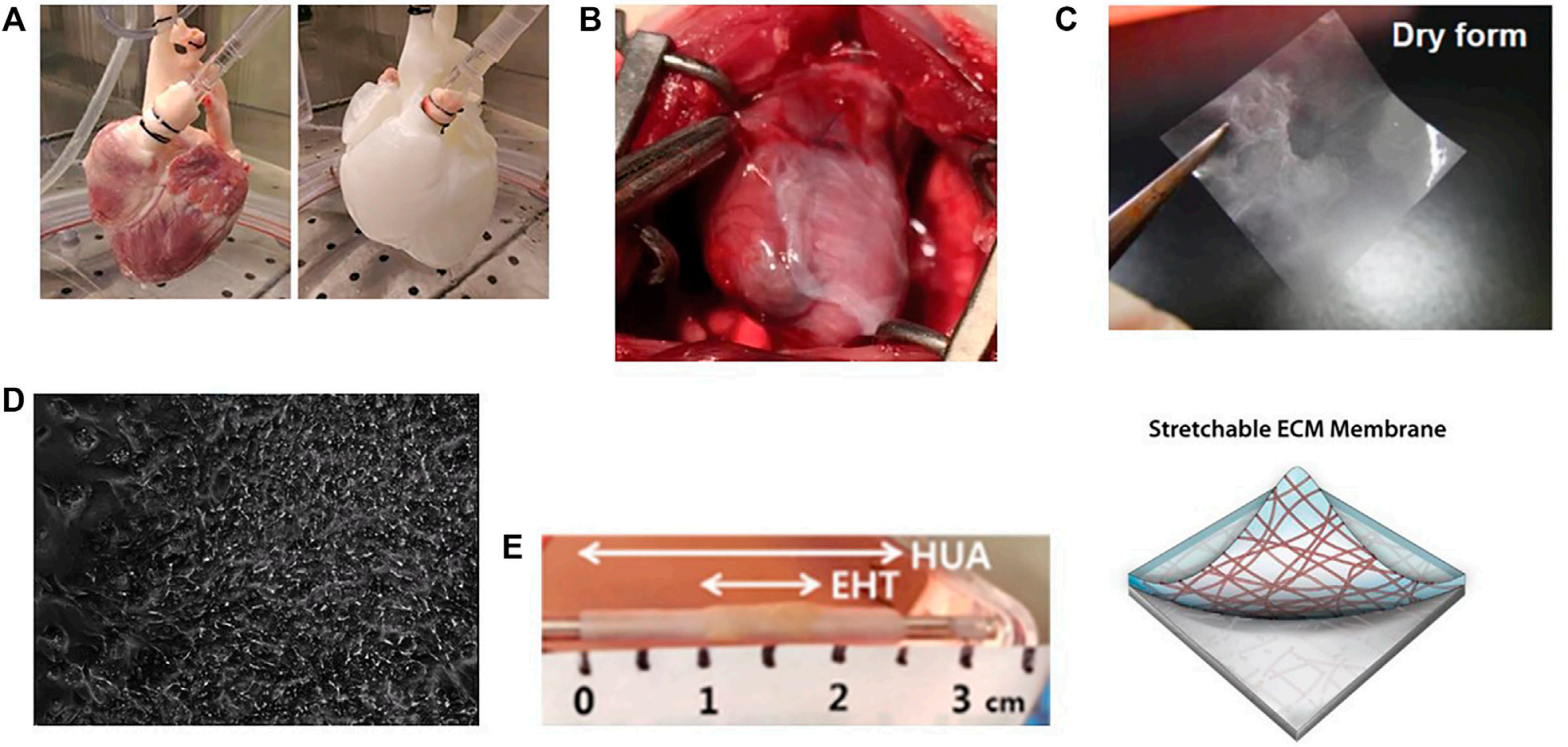
Applications of tissue decellularization for ventricular biofabrication. **(A)** Demonstration of perfusion decellularization of a porcine heart, yielding an acellular extracellular matrix structure retaining the architecture of a heart. Image taken from Tang-Quan et al. (2018). **(B)** Decellularized extracellular matrix can augment ventricular function when applied to damaged myocardium. Image taken from Francisco et al. (2020). **(C)** Decellularized patches can be recellularized and applied to damaged myocardium, not only augmenting function and providing structural support, but also improving angiogenesis and promoting stem cell migration into compromised and damaged ventricular myocardium. Image taken from Kim et al. (2019). **(D)** Decellularized ECM scaffolds recellularized with pluripotent stem cells and programmed to differentiae into ventricular myocardium can display organized contraction. Image taken from Li et al. (2017). **(E)** Beating cardiomyocytes can be seeded onto acellular tubules to generate beating vascular conduits for treatment of congenital ventricular disorders. EHT: Engineered heart tissue (beating cardiomyocytes) seeded onto HUA: decellularized human umbilical artery tissue. Image taken from Kubo et al. (2007).

Tissue decellularization, with or without recellularization, can be applied to augment or fabricate ventricular myocardium (Figure 1). When applied without recellularization, the decellularized extracellular matrix is usually implanted in the form of a patch or a hydrogel into damaged or failing ventricular myocardium to induce regeneration and provide structural support to the myocardium.

### Decellularized Extra-Cellular Matrix

Even in the absence of recellularization, purely decellularized ECM can support ventricular myocardium. Wang et al. minced and decellularized neonatal and adult mice hearts using combination of chemical (antibiotic/antimycotic/gentamycin solution), followed by physical (freezing), and finally enzymatic (deoxyribonuclease and ribonuclease) decellularization, producing an ECM powder that was resuspended into a hydrogel (Wang et al., 2019). They demonstrated that in an adult mouse left anterior descending artery ligation model of myocardial infarction that even a single injection of neonatal mouse decellularized ECM into the ventricular myocardium mitigated left ventricular remodeling, improving ejection fraction, and reducing wall stiffening and fibrosis (Wang et al., 2019). In a cell migration experiment, the group showed that human umbilical vein endothelial cells (HUVECs) demonstrated greater cell activity and migration into neonatal mouse decellularized ECM, postulating that this would lead to downstream angiogenesis (Wang et al., 2019).

Decellularized non-cardiac ECM can be applied in a similar manner to infarcted or damaged ventricular myocardium. Francisco et al. demonstrated that human amniotic membrane could be decellularized via chemical decellularization to produce an ECM bioscaffold patch, impregnated with an anti-inflammatory nanoparticle, and then implanted on the surface of infarcted rat hearts (Francisco et al., 2020). Hearts that received the decellularized amniotic membrane patches, with or without the impregnated nanoparticles, demonstrated greater angiogenesis, decreased scar formation, and greater proliferation of cardiomyocytes, which directly translated into an improved ejection fraction (Francisco et al., 2020).

A limitation to clinical translation of conventional decellularized ECM patches or hydrogels is their physical weakness, ultimately leading to poor long-term engraftment and inability to reliably maintain geometric configurations (Tang-Quan et al., 2018; Kim et al., 2019). Gul Kim et al. offer a potential fix to this issue by stamping decellularized ECM onto polyvinyl alcohol hydrogels, ultimately resulting in a stretchable ECM patch (Kim et al., 2019). When grafted onto infarcted ventricular myocardium, the stretchable decellularized ECM patch not only improved stiffness, ejection fraction, and scar formation, but also improved migration of stem cells into the patched area, with the added three-dimensional structural support that limited priorly described decellularized ECM patches/hydrogels (Kim et al., 2019).

#### Biofabricating Ventricular Myocardium With Recellularization

Bioscaffolds generated from decellularized ECM and then subsequently recellularized stand to provide the benefits of decellularized ECM patches, namely scar limitation, improved left ventricular remodeling and preservation of ejection fraction, with the added potential of repopulating cardiomyocytes. Kajbafzadeh et al. demonstrate this by decellularizing rabbit pericardium and then recellularizing with autologous adipose-derived mesenchymal stem cells (ADMSCs) (Kajbafzadeh et al., 2017; Kameli et al., 2018). Within the patched portions of the ventricular myocardium, the engrafted ADMSCs demonstrated significantly higher populations of CD34 and desmin-positive cells, suggestive of cardiomyocyte regeneration and angiogenesis (Kajbafzadeh et al., 2017; Kameli et al., 2018). Notably, the mesenchymal stem cells within the bioscaffold engrafted beyond the confines of the patch itself, suggesting that these recellularized patches additionally serve as delivery vehicles for stem cell populations (Kajbafzadeh et al., 2017; Kameli et al., 2018).

Tan et al. demonstrated the added benefit of seeding decellularized tissue with stem cells by showing that decellularized small intestinal submucosa patched onto infarcted rabbit ventricular myocardium in itself could preserve ejection fraction and mitigate adverse remodeling, but patches seeded with mesenchymal stem cells grossly improved capillary formation within the infarcted territory of the rabbit ventricular myocardium (Tan et al., 2009). Demonstration of improved neovascularization is a critical step forward in the use of decellularization to address myocardial disease in given that neovascularization is associated with improved survival after ischemic injury to the heart, as demonstrated in long-term studies of coronary artery bypass grafting and basic science investigations of the role of angiogenesis in treating ischemic heart disease (Shi et al., 2021).

Biofabrication of pure ventricular cardiomyocytes for direct engraftment onto damaged ventricular myocardium has been largely limited by challenges in reliably inducing iPSCs to differentiate into ventricular myocardium when delivered via a decellularized patch. Li et al. addressed this challenge by decellularizing adult rat hearts to produce dECM patches, and subsequently recellularizing them with iPSCs genomically engineered through transcription activator-like effector nucleases (TALEN) to select for cues to differentiate into ventricular cardiomyocytes (Garreta et al., 2016; Li et al., 2017). The result of this decellularization-recellularization method was functional, electrically conductive, beating ventricular myocardium in a petri dish (Li et al., 2017). Though this myocardium has not been grafted onto hearts, reliably converting iPSCs into fully differentiated myocardium represents a major step forward in clinical and therapeutic translation of these methodologies.

Even without directly fabricating ventricular myocardium, decellularization and recellularization can improve currently existing therapies for ventricular disease. For example, congenital defects that result in single ventricle physiology can be treated using surgical techniques such as the Fontan procedure. This procedure utilizes a vascular conduit to redirect central venous blood flow directly into the pulmonary artery in situations where the right ventricle is physiologically or physically absent. A struggle of the procedure is reliably generating forward flow into the pulmonary arterial circuit. Park et al. demonstrate a novel technique to generate a pulsatile vascular conduit that can act like the right ventricle to generate stroke work to push blood flow into the pulmonary artery circuit (Shi et al., 2021). The approach was two-fold: 1) generate a beating sheet of cardiac myocytes, and 2) generate a conduit onto which to wrap the beating cardiomyocytes. The former was produced by chemically decellularizing porcine hearts and recellularizing with a mix of iPSCs with commercially available adult cardiac fibroblasts. The latter was achieved by decellularizing human umbilical cord. The muscle sheets were then engrafted onto the decellularized cord, ultimately producing a pulsatile conduit that could be used for alleviation of congenital single ventricle disease (Park et al., 2020). This approach radically improved on prior attempts at producing pulsatile conduits mainly through its use of decellularization. Prior work has utilized fibrin scaffolds instead of decellularized umbilical cord as the conduit, producing a much smaller amount of stroke work compared to the method developed by Kubo et al. (2007); Park et al. (2020).

#### Limitations and Future

Ventricular biofabrication represents an area of application of decellularization/recellularization that has not been meaningfully translated into clinical practice, unlike other applications such as bovine pericardial patches or certain decellularized heartvalves (Tang-Quan et al., 2018). Though conventions have been accepted for levels of DNA present in decellularized tissue, the ability to recellularize this tissue and produce functioning myocardium is dependent on preservation of the microarchitecture of the ECM, including appropriate amounts and arrangement of mature, cross-linked collagen, glycosaminoglycans, and growth factors (Kc et al., 2019). The various decellularization strategies described previously may disrupt this microarchitecture, and substantial heterogeneity exists in choosing not only the optimal method, but controlling each of the parameters within each method, be it perfusion, mechanical, or enzymatic decellularization. In addition to removing adequate DNA, while preserving the vital components of the ECM, decellularization must minimize the presence of immunogenic factors such as human leukocyte antigens when using human hearts for decellularization or immunogenic sugars in xenogeneic hearts, an area of active investigation (Galili, 2015; Guyette et al., 2016; Kc et al., 2019). Finally, when decellularizing tissue that has sustained damage whether by decellularization or by the natural history of the heart, there are structural changes in the microvasculature that may hinder recellularization due to disruption of perfusion networks (Guyette et al., 2016).

Preserving the ECM microarchitecture contributes significantly to the major limitation of recellularization: generating enough viable cardiomyocytes from stem cells. Several billion, organized and functioning cardiomyocytes are needed to repopulate a whole decellularized human heart (Guyette et al., 2016). While patches of recellularized dECM have shown potential to augment cardiac function, whole organ or specific ventricular replacement requires highly organized cardiomyocyte electrical, mechanical, and paracrine function. Having enough stem cells to mature and populate a decellularized cardiac graft is incumbent on successful implantation of stem cells, followed by ideal ECM microenvironment conditions to promote appropriate differentiation into the appropriate cell lines (Brizzi et al., 2012; Kc et al., 2019). Current recellularization methods are limited in appropriately distributing multiple stem cell lines to promote not only survival but appropriate differentiation into the organized subunits necessary for complex cardiac function (Vunjak-Novakovic et al., 2010; Kc et al., 2019). However, since 2016 there has been an explosion of meaningful advancements to overcoming the challenges facing ventricular biofabrication. These are namely, the reliable programming of induced pluripotent stem cells into cardiomyocytes, utilizing genomic engineering or optimized cell culture protocols, and clever decellularization of non-cardiac tissue ultimately for cardiac applications. One such advancement in improving stem cell survival and functional maturation is recognition and use of organized mechanical stimuli through biomimetic bioreactors to support recellularization (Hülsmann et al., 2013; Wang et al., 2013; Guyette et al., 2016). Bioreactors are used to simulate contraction of the decellularized cardiac graft while recellularization is ongoing, and the current era of these bioreactors are highly customizable, and have improved the survival and maturation of cardiomyocytes in decellularized human hearts (Guyette et al., 2016).

The clinical utilization of these fabricated sections of ventricular myocardium will likely be incumbent on the reliable generation of microvasculature and generation of a reliable conduction system as well.

## Conclusion

Biofabrication of ventricular myocardium has been propelled forward, especially in the past 6 years, through the advent of decellularization and recellularization techniques. Though there are competing methodologies to produce functional myocardium or augment dysfunctional myocardium, decellularization based methods uniquely provide organized, three-dimensional structural support, while preserving biophysical cues to instruct native or seeded stem cells to promote angiogenesis and differentiation into fully functional cardiomyocytes. The therapeutic potential of biofabricated myocardium stands to revolutionize the treatment of ischemic cardiomyopathy, and the last decade has introduced unique advancements in these methodologies, bringing the field closer to meaningful clinical translation.
